# Trends of admissions and case fatality rates among medical in-patients at a tertiary hospital in Uganda; A four-year retrospective study

**DOI:** 10.1371/journal.pone.0216060

**Published:** 2019-05-14

**Authors:** Robert Kalyesubula, Innocent Mutyaba, Tracy Rabin, Irene Andia-Biraro, Patricia Alupo, Ivan Kimuli, Stella Nabirye, Magid Kagimu, Harriet Mayanja-Kizza, Asghar Rastegar, Moses R. Kamya

**Affiliations:** 1 Department of Medicine, Makerere University College of Health Sciences, Kampala, Uganda; 2 Department of Medicine, Uganda Cancer Institute, Kampala, Uganda; 3 Department of Medicine, Yale School of Medicine, New Haven, Connecticut, United States of America; 4 Department of Medicine, Makerere Lung Institute, Kampala, Uganda; 5 Directorate of Medicine, Mulago National Referral Hospital, Kampala, Uganda; Azienda Ospedaliero Universitaria Careggi, ITALY

## Abstract

**Background:**

Sub-Saharan Africa suffers from a dual burden of infectious and non-communicable diseases. There is limited data on causes and trends of admission and death among patients on the medical wards. Understanding the major drivers of morbidity and mortality would help inform health systems improvements. We determined the causes and trends of admission and mortality among patients admitted to Mulago Hospital, Kampala, Uganda.

**Methods and results:**

The medical record data base of patients admitted to Mulago Hospital adult medical wards from January 2011 to December 2014 were queried. A detailed history, physical examination and investigations were completed to confirm the diagnosis and identify comorbidities. Any histopathologic diagnoses were made by hematoxylin and eosin tissue staining. We identified the 10 commonest causes of hospitalization, and used Poisson regression to generate annual percentage change to describe the trends in causes of hospitalization. Survival was calculated from the date of admission to the date of death or date of discharge. Cox survival analysis was used to identify factors associate with in-hospital mortality. We used a statistical significance level of p<0.05. A total of 50,624 patients were hospitalized with a median age of 38 (range 13–122) years and 51.7% females. Majority of patients (72%) had an NCD condition as the primary reason for admission. Specific leading causes of morbidity were HIV/AIDS in 30% patients, hypertension in 14%, tuberculosis (TB) in 12%), non-TB pneumonia in11%) and heart failure in 9.3%. There was decline in the proportion of hospitalization due to malaria, TB and pneumonia with an annual percentage change (apc) of -20% to -6% (all p<0.03) with an increase in proportions of admissions due to chronic kidney disease, hypertension, stroke and cancer, with apc 13.4% to 24%(p<0.001). Overall, 8,637(17.1%) died during hospitalization with the highest case fatality rates from non-TB pneumonia (28.8%), TB (27.1%), stroke (26.8%), cancer (26.1%) and HIV/AIDS (25%). HIV-status, age above 50yrs and being male were associated with increased risk of death among patients with infections.

**Conclusion:**

Admissions and case fatality rates for both infectious and non-infectious diseases were high, with declining trends in infectious diseases and a rising trend in NCDs. Health care systems in sub-Saharan region need to prepare to deal with dual burden of disease.

## Introduction

The disease burden in developing countries is continuing to grow faster than the budget allocation for healthcare. In low income countries (LIC) medical admissions account for about 40% of total hospital admissions compared to 12–30% in high income countries[1,2]. This may be a reflection of disparities in socioeconomic conditions and healthcare systems, or differences in biological and/or environmental factors[3–5]. One factor which exacerbated the healthcare crisis in sub-Saharan Africa (SSA) was the advent of the HIV/AIDS epidemic which disproportionately affects the region [2,6,7]. Interventions, such as antiretroviral therapy for the treatment of HIV/AIDS, health education, access to clean water, and mass vaccinations, however, have decreased morbidity and mortality from communicable diseases[8]. The improved life expectancy and adoption of western lifestyles in LICs have led to the emergence of non-communicable diseases (NCDs), a new threat to public health[9,10].

In Uganda as with other sub-Saharan African countries, there is an emerging increase in NCDs such as hypertension and diabetes mellitus, impacting the health of patients with and without HIV[11–14]. However, as modeling studies show, malaria and lower respiratory infections are the leading causes of years of life lost[15]. Despite the growing evidence of declining trends of HIV in the community[16], anecdotal observations in Mulago hospital indicate that HIV-AIDS is still the leading cause of morbidity and mortality on medical wards in Uganda[17]. Few studies have examined the major causes of morbidity and mortality in Uganda and most of them have focused on individual diseases[17–20]. An autopsy study in a predominantly HIV-infected population on Mulago hospital carried out on 2 medical ward units only (Infectious diseases and Gastro-intestinal) noted that the leading cause of death among HIV-infected patients were tuberculosis and cryptococcal meningitis[21]. In contrast, in patients without HIV infection, the leading causes of death were non-infectious, with upper gastrointestinal bleeding secondary to liver cirrhosis being the most common[21]. Since 2007, Mulago Hospital has been operating specialty-based in-patient services run by sub-specialist physicians. There is however, limited data on trends in the incidence of specific diseases, as well as morbidity and mortality in these specialized units. Understanding the major drivers of morbidity and mortality in one of the largest hospitals in the region would help inform health systems improvements for Uganda and the East African region[22].

We therefore sought to describe the leading causes of hospitalization and death on the medical wards among adult patients in Mulago National Referral Hospital, Kampala, Uganda.

## Materials and methods

We conducted a retrospective cohort study of patients admitted to Mulago National Referral Hospital adult medical wards from January 2011 to December 2014 using an electronic patient data registry. Mulago National Referral Hospital is one of the two national referral hospitals in a country with a population close to 40 million. It has 1500 bed-capacity and provides specialized care for patients referred from district and regional referral hospitals. In 2007, the Directorate of Medicine established sub-specialist run units to cater to the increasing specialized care needs. Consultants, physicians and senior house officers run these units. Patients undergo preliminary evaluation and investigations in the accident and emergency unit before admission to the appropriate specialized unit. On average, 15,000 emergency medical visits are made to Mulago National Referral Hospital annually and between 900–1200 patients are admitted to the medical wards per month[17].

Each patient receives a detailed history, physical examination and investigations are completed to confirm the diagnosis and identify comorbidities. Investigations performed include a complete blood count, HIV testing, chemistry, abdominal ultrasound, chest x-ray, sputum gene-expert, bone marrow biopsy, lumbar puncture, echocardiogram, and electrocardiogram as clinically indicated. Any histopathologic diagnoses were made by hematoxylin and eosin tissue staining.

In 2010, a patient registry was established in the Department of Medicine through the Rainer Arnhold Senior House Officers’ Teaching Support (RASHOTS) project. The purpose of the database was to support evaluation and improvements in quality of patient care and training for postgraduate students of Makerere University College of Health Sciences.

Three trained medical records clerks capture information on patient’s unique number, age, gender, district of residence, diagnosis, and vital status from the patient files at discharge. They enter this information directly into a password protected access registry. The diagnosis captured by the data clerks is the final diagnosis in the chart at the time of discharge or death. Diagnosis is coded based on the tenth revised International Classification of Diseases (ICD-10) [23]. Age groups were defined based on the World Health Organization "Provisional guidelines on standard international age classification” available at https://unstats.un.org/unsd/publications/. We used the ward/ service of admission as proxy for the reason for admission. Patients on cardiology, nephrology, neurology, and hematology were considered to have non-communicable disease (NCD) as the primary reason for admission whereas patients on infectious diseases wards and pulmonology (admits primarily pneumonia and TB patients) were considered to have an infectious disease (ID) as the primary reason for admission.

For this study, de-identified patient information was exported into excel using a standardized approach. We excluded cases with missing information on diagnosis and date of admission. Summary statistics were reported using medians and ranges for continuous variables and frequency counts and percentages for categorical variables. We identified the 10 commonest causes of hospitalization, and used Poisson regression to generate annual percentage change to describe the trends in causes of hospitalization. We used Pearson chi-square test for categorical variable and Wilcoxon rank-sum test for continuous variables to assess association between variables. Survival was calculated from the date of admission to the date of death or date of discharge. Cox survival analysis was used to identify factors associate with 30-day mortality. We used a statistical significance level of p<0.05. All analyses were performed using STATA version 13 (STATA Corp, College Station, TX).

The study was approved and received a waiver of consent from the Makerere University School of Medicine Research Ethics Committee and Uganda National Council of Science and Technology (UNCST).

## Results

**Fig 1:**
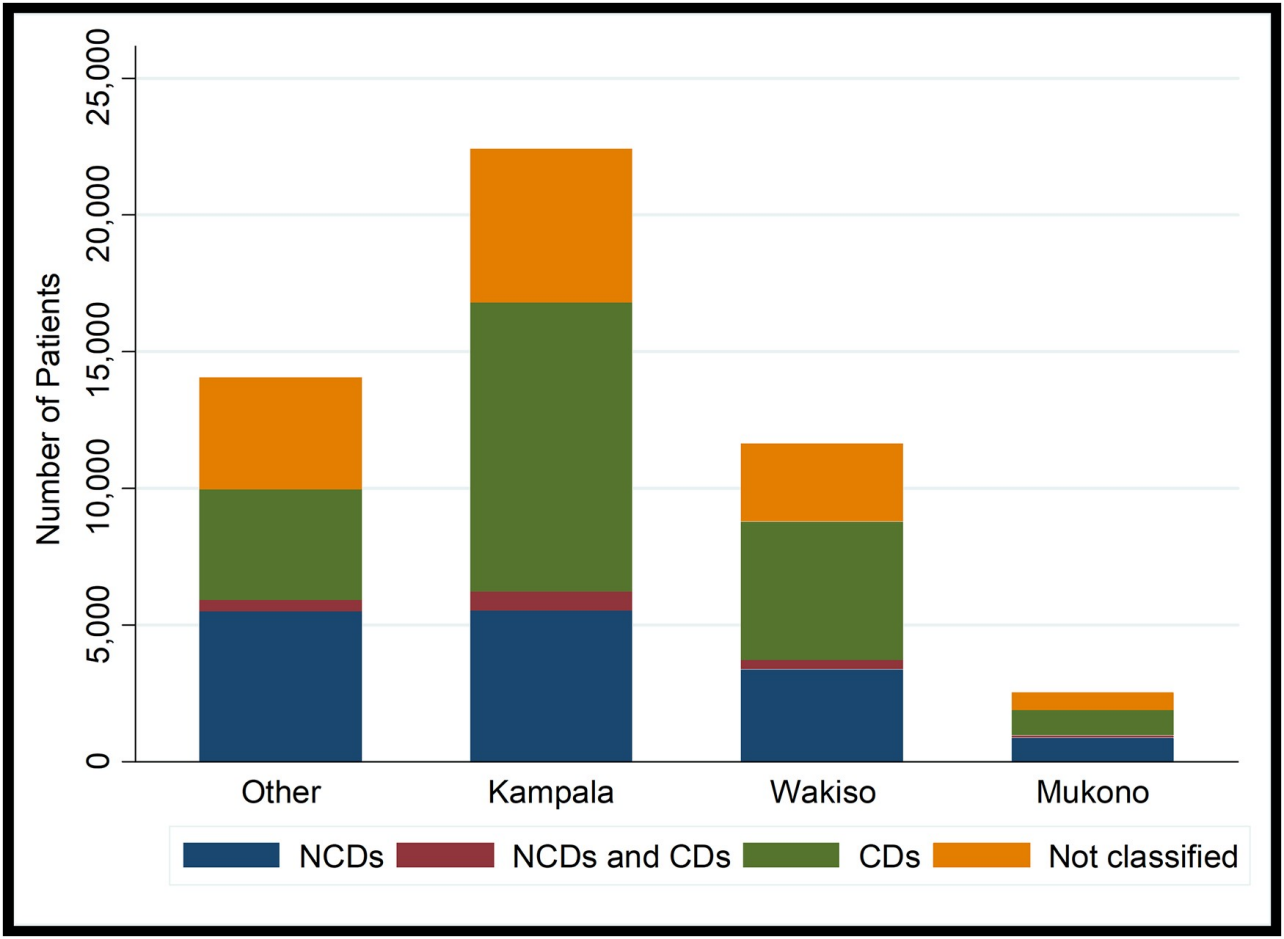
Distribution of diagnoses by district of residence among patients admitted to Mulago Hospital. Abbreviations: CDs-Communicable disease; NCDs-Non-communicable disease.

During the study period, there were 50,716 patient hospitalizations. We excluded 92 for lack of complete admission date. Of the remaining 50,624 patients, 23% were admitted in 2011, 27.2% in 2012, 25.7% in 2013, and 24.1% in 2014, 51.7% of these patients were female. The median age was 38 years (range13-122) About two thirds of patients were residents of urban and periurban areas (Kampala Capital City and its suburbs) (Fig 1 and Table 1).

**Table 1:** Patient characteristics overall and by year of hospitalization at Mulago Hospital.

Characteristic	Overall (N = 50,624)	2011 (N = 11,637)	2012 (N = 13,761)	2013 (N = 13,028)	2014 (N = 12,200)	P value
**Age, median(range)**	38(13–122)	38(13–122)	37(13–122)	38(13–110)	40(133–120)	0.001
**Age group, n (%)**						
**13–24**	9,135(18.0)	2,126(18.3)	2,567(18.7)	2,393(18.4)	2,049(16.8)	0.001
**25–54**	28,866(57.0)	6,619(56.9)	8,055(58.5)	7,322(56.2)	6.870(56.3)	
**55–74**	8,574(16.9)	2,037(17.5)	2,141(15.6)	2,230(17.1)	2,166(17.8)	
**>74**	3,716(7.3)	771(6.6)	925(6.7)	985(7.6)	1,035(8.5)	
**Female, n (%)**	26,175(51.7)	6008(51.7)	7,134(51.9)	6,709(51.5)	6,324(51.9)	0.9
Urban residence*	36,469(72.0%)	7,978(68.6)	10,100(73.4)	9,497(72.9)	8,894(72.9)	0.001

*Urban–included Kampala City and its suburbs (Wakiso and Mukono districts)

Patients in the age bracket of 25–54 years accounted for over half of the admissions overall and across the years studied. Patients admitted in the later years of the study were significantly older compared to the earlier years of the study (p = 0.001). There was no difference in gender distribution of patients across the study period (0.11).

The primary reason for admission was an NCD condition in 62% of patients. Patients on the NCD wards were significantly older than patients on the ID wards (median age 42 vs 35 years, p<0.001), and more likely to be female (53% vs 49%, p<0.001). The leading NCDs included hypertension in 14.2% of the patient population, diabetes in 7%, heart failure in 9.3%, stroke in 4.9%, anemia in 7%, and cancer in 3.9%. Similarly, the leading infectious disease conditions included HIV in 30.4% of patients, tuberculosis (TB) in 13.1%, non-TB pneumonia in 11.1%, malaria in 6.9%, gastroenteritis in 4 and sepsis in 4.26%. HIV co-infection was 14.2% on the NCD wards compared to 57.6% on the ID wards. The commonest AIDS defining conditions included tuberculosis in 31.6% of patients with HIV, cryptococcal meningitis in 7.7%, KS in 2.1%, and PCP in 1.5%.

**Fig 2:**
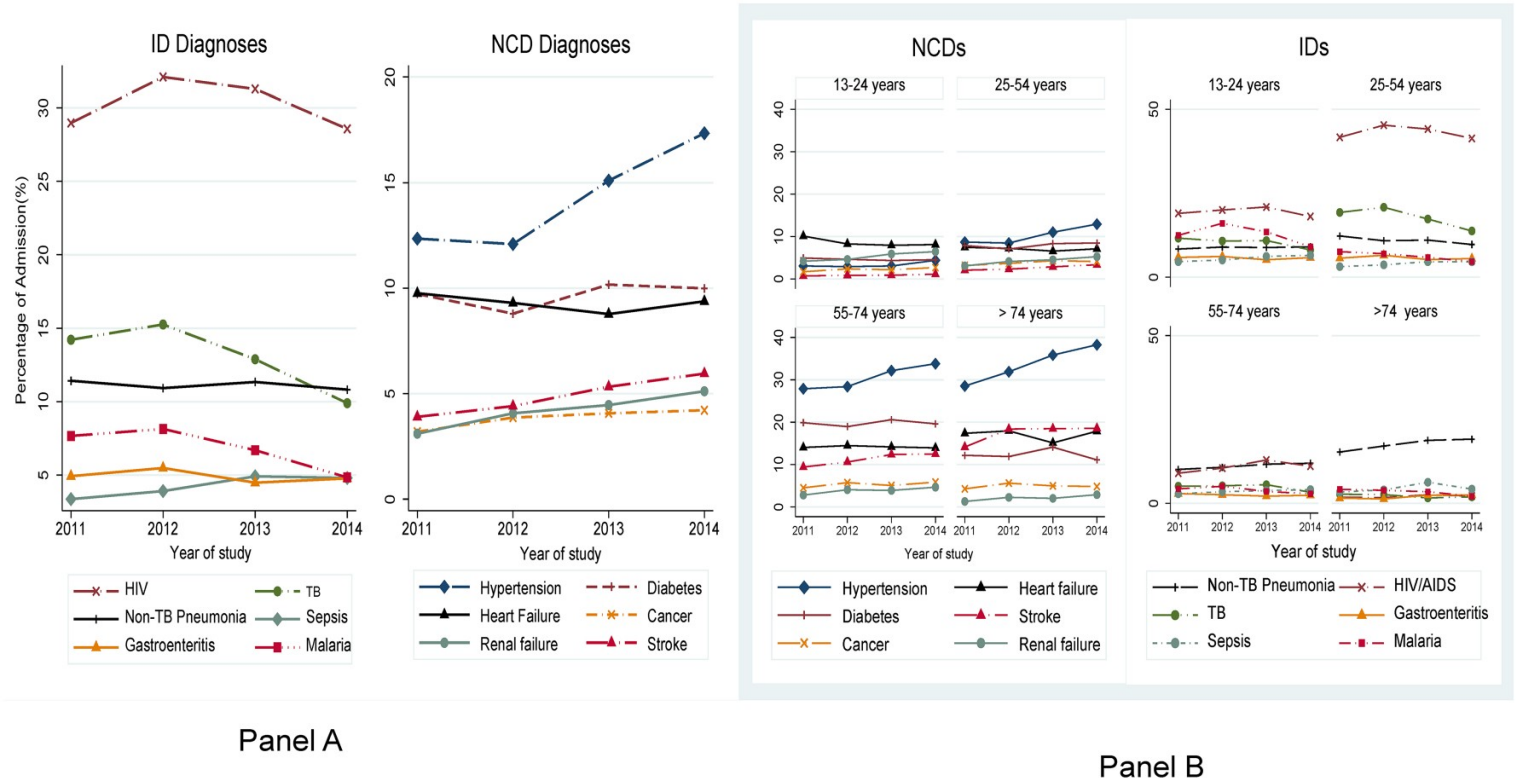
Trends in major causes of morbidity by age group and calendar year at Mulago Hospital, 2011–2014. Panel A shows trends of communicable and non-communicable diseases through the four years of study. Panel B shows the major causes of death by age group through the four years of study. **Abbreviations**: CDs-Communicable disease; NCDs-Non-communicable disease.

There was a general downward trend in communicable disease diagnoses and an upward trend in in NCD diagnoses over the study period (Fig 2 panel A). There was a significant decline in the proportion of patients with a final discharge diagnosis of malaria with an apc of -20% (95% CI: 0.71–0.91, p = 0.001), TB with an apc -12% (95% CI: 0.78–0.98, p = 0.03), and pneumonia with an apc of -6% (95% CI: 0.89–0.99, p = 0.02). In contrast, there was a significant increase in the proportion of patients with a final discharge diagnosis of CKD (apc = 24%, 95% CI:1.18–1.31, p<0.001), hypertension (apc = 14% (95% CI:1.11–1.16, p <0.001), stroke (apc = 13.4%(95% CI: 1.09–1.18), cancer (apc = 10%, 95% CI: 1.06–1.15, p<0.001), and heart failure (apc = 6%, 95% CI:1.04–1.09, p<0.001). However, there was no significant change in the number of patients with admission diagnosis of HIV/AIDS (p = 0.8) and diabetes mellitus (p = 0.09) (Fig 2 panel B).

The distribution of these common conditions was markedly different among the four age groups is shown in Fig 2. NCDs predominated in the older age groups (above 54 years) compared to IDs in younger age groups (54 and below). Among those aged 25–54 years the prevalence of HIV was 43.2% compared to 10.9% in those 55–74 years of age; Tb was at 17.9% compared to 4.8%. On the contrary hypertension was commoner in those above 54 years of age (30% in 55–74 years and 34% in those 75 years and above) compared to 10.2% among those 25–54 years of age. The highest median age was 64 years (IQR: 50–75) among stroke patients and 57 years (IQR: 43–70) for hypertension, and the lowest median age was 30 years (IQR: 21–42) for malaria. Across the entire population, HIV infected patients were younger than those who were HIV negative (median age 41 years [IQR: 27–60] vs 35years [28–42], p <0.001).

Women constituted the majority of patients for all diagnoses except for TB (44%) and cancer (43.1%) and constituted about half for non-TB pneumonia (50%), and CKD (52.2%). Additionally, there was a greater proportion of HIV-infected women than men (50.1% vs 54%).

**Fig 3:**
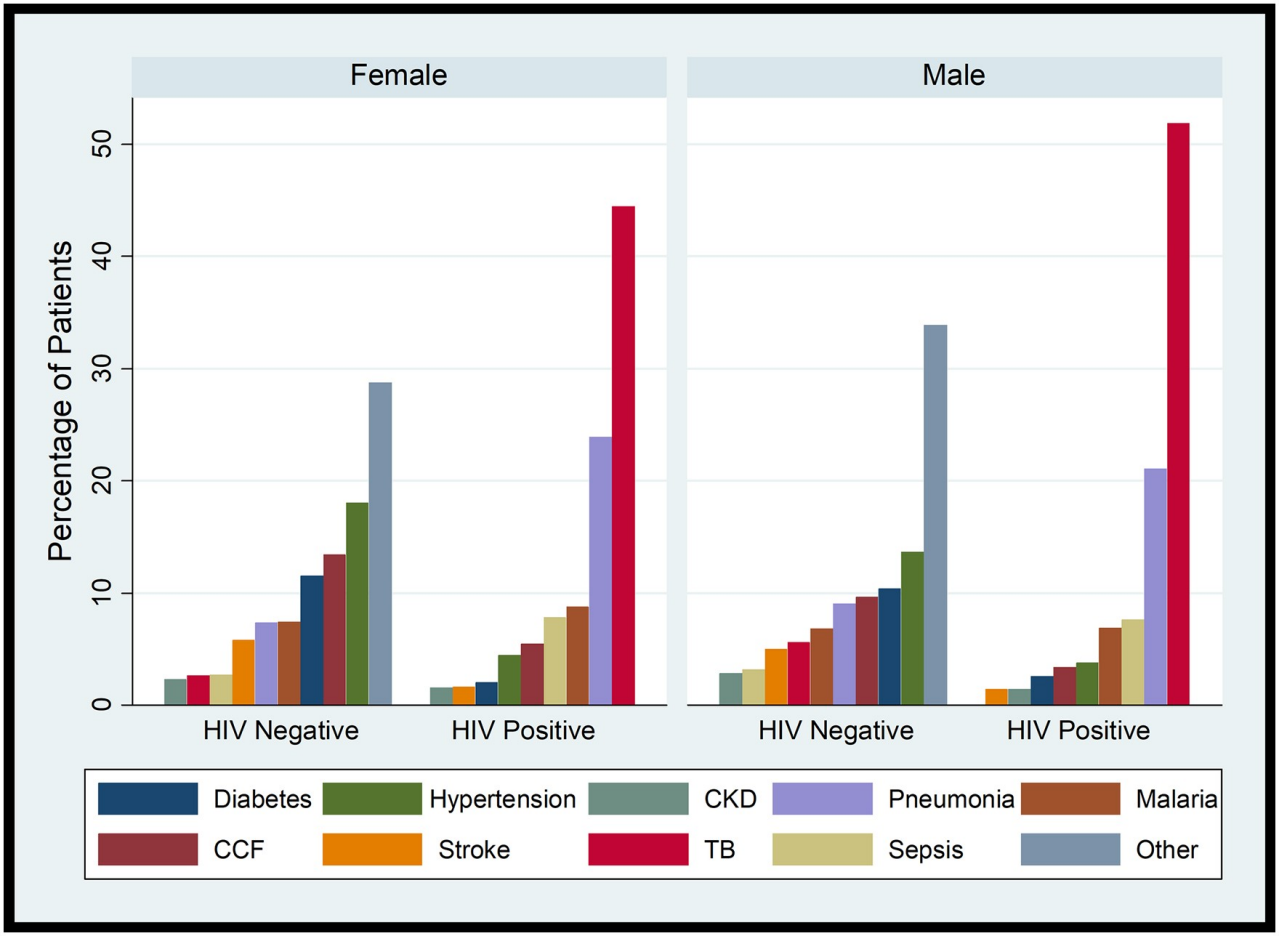
Distribution of discharge diagnoses by gender and HIV status among patients in Mulago Hospital. **Abbreviations**: CCF: Congestive cardiac failure; CKD-chronic kidney disease; TB-tuberculosis.

HIV comorbidity was highest among patients with discharge diagnoses of TB (72.9%) and non-TB pneumonia (38.4%), and lowest among patients with discharge diagnoses of diabetes (4.5%) and hypertension (5.5%) (Fig 3).

**Fig 4:**
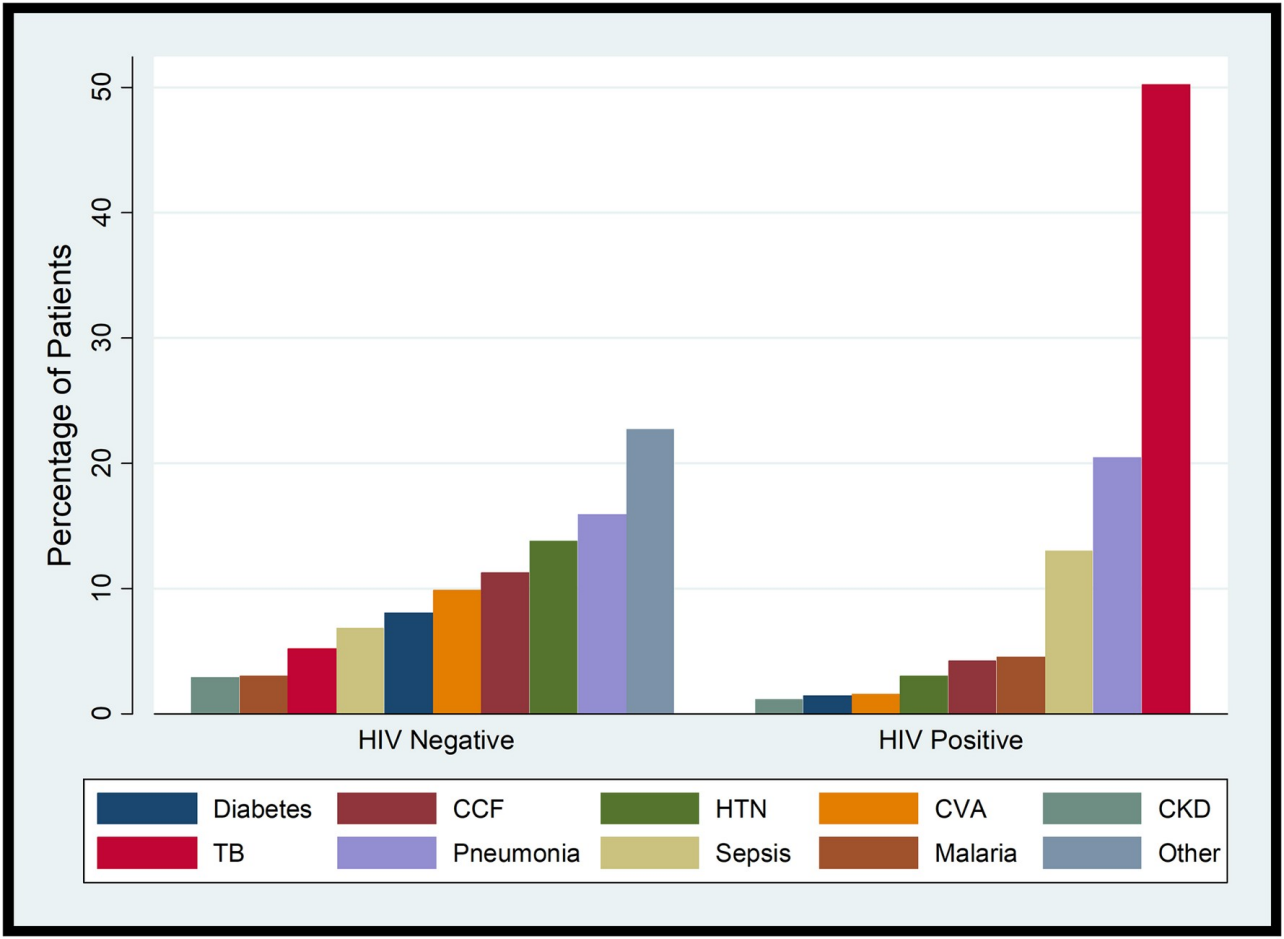
Distribution by HIV Serostatus of discharge diagnoses among patients who died during hospitalization. Abbreviations: CCF-Congestive cardiac failure; CKD-chronic kidney disease; CVA-cardiovascular accident; HTN-Hypertension; TB-tuberculosis.

Overall 8,637(17.1%) died during hospitalization; the proportion of patients who died during hospitalization increased over the period from 13.1% in 2011, 18% in 2012, 18.7% in 2013 and 18.3% in 2014. Among the patients who died during their hospitalization, the final diagnoses listed in the chart included HIV/AIDS in 44.5%, TB in 19.7%, non-TB pneumonia in 17.9%, sepsis in 9%, malaria in 3.6%, hypertensions in 10.8%, diabetes in 6.2%, heart failure in 9.4%, CVA in 7.6%, CKD in 2.45%, and cancer in 6.4% (Fig 4).

In multivariate analysis, age, gender, HIV status, and district of residence were all associated with in-hospital mortality. Increasing age was significantly associated with increased risk of in-hospital mortality, with an increase of 7% among patients 31–60 years of age and 46% among patients with >60 years of age, compared to those less than 30 years of age(p = 0.01). Similarly, males had 23% increased risk of death compared to females (p <0.001). Patients with HIV had a 68% increased risk of death compared to their HIV negative counterparts (p<0.001). Compared to the baseline year (2011), the risk of death was significantly higher in all subsequent years–increased by 24% in 2012, 28% in 2013, and 30% in 2014 (Table 2). Patients with HIV where more likely to have non-TB pneumonia than their none HIV-infected counterparts irrespective of sex.

**Table 2:** Factors associated with. in-hospital mortality among patients admitted to Mulago Hospital.

Characteristic	Univariate Analysis		Multivariate Analysis	
	HR(95% CI)	p	HR(95% CI)	p
**Age category**				
**11–30**	**ref**		**ref**	
**31–50**	**1.12(1.07–1.18)**	**< 0.001**	**1.07(1.02–1.23)**	**0.001**
**>50**	**1.21(1.13–1.29)**	**< 0.001**	**1.46(1.37–1.56)**	**< 0.001**
**Gender, n (%)**				
**Female**	**ref**		**ref**	
**Male**	**1.21(1.15–1.26)**	**< 0.001**	**1.23 (1.17–1.28)**	**< 0.001**
**District of residence**				
**Others**	**ref**		**ref**	
**Kampala**	**1.10(1.04–1.16)**	**0.001**	**1.05(0.99–1.11)**	**0.08**
**Wakiso**	**1.19(1.12–1.27)**	**<0.001**	**1.13(1.06–1.20)**	**<0.001**
**Mukono**	**1.35(1.23–1.49)**	**<0.001**	**1.30(1.17–1.43)**	**<0.01**
**Year of hospitalization**				
**2011**	**ref**		**ref**	
**2012**	**1.10(1.04–1.16)**	**< 0.001**	**1.24(1.16–1.33)**	**< 0.001**
**2013**	**1.19(1.12–1.27)**	**< 0.001**	**1.29(1.20–1.38)**	**< 0.001**
**2014**	**1.35(1.23–1.49)**	**P < 0.001**	**1.30(1.22–1.40)**	**P < 0.001**
**HIV-coinfection, n (%)**				
**Negative**	**Ref**		**ref**	
**Positive**	**1.55(1.48–1.62)**	**< 0.001**	**1.68(1.60–1.76)**	**< 0.001**

## Discussion

In this retrospective cohort study of patients admitted to Mulago Hospital adult medical wards we found the leading diagnoses among hospitalized patients to be infections with HIV-AIDS, Tuberculosis and Malaria. The leading NCD diagnoses among hospitalized patients were hypertension, heart failure and diabetes mellitus.

Overall, the leading final diagnoses among patients who died in the hospital were HIV-AIDS and Tuberculosis for infections, and hypertension and heart failure for NCDs. There was a decline in percentage of hospitalization from infectious diseases over the four years with proportional increase in non-communicable diseases. The case fatality rate for communicable diseases was highest in patients with final diagnoses of non-TB pneumonia, TB, and HIV-AIDS while among patients with NCD final diagnoses, the case fatality rates were highest in those with stroke, cancer and chronic kidney disease. In-hospital mortality rates increased over the four years of the study.

These findings are similar to what has been reported in other parts of Africa. A study from Cameroon found the leading cause of death among hospitalized patients was related to HIV-AIDS[24]. In Malawi, among 2,911 of patients admitted in Kamuzu Central Hospital medical wards, up to 81% were HIV positive. Being HIV-positive, antiretroviral therapy (ART)-naïve or being a new ART-initiator were associated with high risk of mortality compared to HIV-negative patients[25].

Another study from South Africa found a high mortality rate in patients admitted to the medical wards with an inpatient mortality of 11% with a 12-month mortality of 41%[26]. Age above 40 years and a urea level above 7.0mmol/l, a diagnosis of HIV, TB or sepsis were associated with poor outcomes at 12 months. These findings serve to show the large contribution of infections to mortality, particularly in LICs, as has been outlined in the global burden of disease mapping[15]. It is quite surprising that ‘the big three’ diseases that have received most attention across the globe still remain a major cause of morbidity in Uganda while global trends are showing a decline for HIV and malaria[27,28].

Though we found declining trends of admissions from infectious diseases compared to NCDs, this has not been the case in other countries. As an example, in one South African study, the rates of admission increased from 228 to 628 over a similar 2-month period in 10 years (1991 and 2002) with tuberculosis and lower respiratory tract infections being the leading causes of admissions[29]. The reduction in rates of admission could be mirroring the improved care and survival of patients with HIV and its associated complications due to increased access to care and presence of robust ambulatory health systems for care of these patients[22,30].

On the flipside, few studies have described morbidity and mortality from NCDs as a whole. In a systematic review and meta-analysis of studies of patients with heart failure in LIC, the major cause of heart failure was hypertension among patients from African countries compared to their counterparts from other LICs where the leading cause of heart failure was ischemic heart disease[31]. The current literature largely focuses on specific NCDs among admitted patients. In one study in central Ghana, NCDs were the leading cause of death among patients hospitalized with neurological disorders[32]. In that study, the commonest cause of admission was cardiovascular accidents found in 54% of patients, increasing age was associated with higher mortality with a hazard ratio of 1.11(CI95% 1.06–1.17) for each 20-year increase in age. In the sub-Saharan Africa Survey of Heart Failure (THESUS-HF) study looking at acute heart failure from 12 hospitals in 9 SSA countries, the main predictors of 180-day mortality were malignancy, severe lung disease, smoking history, systolic blood pressure, kidney dysfunction, anaemia, and being HIV-positive[33]. In Mbarara Hospital in Eastern Uganda, patients who were hospitalized with heart failure had a doubled risk of death compared to their counterparts with heart failure who were not hospitalized for heart failure exacerbations after 6 months of follow up[34]. Another study demonstrated that Glasgow coma score of less than 14 and renal failure were major predictors of mortality among admitted patients[35]. There are few studies looking at trends of admissions among patients admitted with NCDs. A study from Nigeria showed that in the medical wards, the number of admissions from NCDs was 2 times that of infectious diseases[36]. A systematic review of 86,307 admissions to medical wards in Africa suggests that although infections are still the leading cause of admissions, cardiovascular diseases increased by fivefold from 3.9% (1950–59) to 19.9% (2000–2010) RR 5.1 (95% CI 4.5–5.8, test for trend p<0.00005)[37]. This trend seems to be similar to our findings.

There is an increasing interest in the overlap between infections and non-communicable diseases with particular attention to HIV. Though the patients with final diagnoses of DM in our study had very low rates of HIV co-infection, there has been a great overlap between HIV, TB and diabetes from studies done in other parts of Africa[38]. Diabetes mellitus increases the risk of active TB regardless of study design and population[39]. Patients who have diabetes with TB have increased risk of adverse outcomes such as treatment failure, TB relapse as well as death[39,40]. This calls for combined efforts in fighting both NCDs and infections rather than dealing with them as separate diseases beginning with the ambulatory clinics[13,41–44].

In our population, non-TB pneumonia, TB, HIV-infection, stroke, cancer and chronic kidney diseases were the diagnoses with the highest case fatality rates. It is hard to explain why non-TB pneumonia has the highest case fatality rate among our patients. This may be due to the fact that patients report late to hospital and may need oxygen and respiratory support which is not readily available, coupled with high rates of antibiotic resistance and frequent drug stock-outs at the hospital[20,37,45,46]. That said, patients with stroke, cancer or kidney disease often report with advanced disease to the hospital and there is lack of advanced healthcare services available for their care and support[47–50]. Another reason may be HIV-associated infection. Non-TB pneumonia was higher in HIV-infected patients compared to HIV negative patients regardless of gender.

The in-hospital mortality rates increased over the four years in comparison to 2011. This is rather surprising and may need further exploration to look at the driving factors of mortality which may be beyond the scope of this study.

Our study had several strengths. First, we looked at all the patients admitted within the study period with only less than 0.1% failing to meet the inclusion criteria. Additionally, to our knowledge, this is the first study looking at admissions, discharge diagnoses, and trends in mortality over a large category of diseases in the largest hospital in Uganda. As a result, we have been able to demonstrate that, though infectious diseases are still the leading cause of morbidity and mortality on the medical wards, NCDs are rising as a major cause of admissions and death. The healthcare system needs to adopt to this new change and provide resources to ensure that the health care system as a whole is able to deal with this rising tide[51,52].

Our study also had several weaknesses. The retrospective nature of the study means limited access to other factors that may predict outcomes, such as vital and other clinical signs at the time of presentation to the hospital, as well as laboratory results. We used the primary diagnosis as was made by the treating team at discharge and did not capture deaths on arrival as well as re-admissions. We also acknowledge the limitation of confirming a diagnosis in our patient population. These factors may have underestimated the rate and contributions of other co-morbidities to illness and mortality. Co-morbidities were only identified if they were included on the discharge form. The diagnosis that occurred in lower proportions were not reflected as specific diagnoses. We were also not able to capture data on surgical causes of morbidity and mortality because these are admitted to the surgical wards independent of the medical wards of our study focus. Additionally, there are two private subspecialized units on the Mulago campus (the Uganda Heart Institute and the Uganda Cancer Institute) where patients with cardiovascular disease and cancer can be referred or directly admitted. As these units were not included in our data collection, this may have led to an under-estimation of these two diseases in our study. However, the RASHOTS team undertakes due diligence to ensure that all patients admitted to the hospital had their data captured to the greatest detail available.

## Conclusion

We found the leading discharge diagnoses in a national referral hospital to be HIV-infection, hypertension, tuberculosis and heart failure. Among all of the characterized diagnoses, non-TB pneumonia, TB, stroke, cancer and chronic kidney disease had the highest case fatality rates. There is an increasing trend of NCDs as a major cause of admissions over the 4-year period of study. There is an urgent need for Ugandan health care system to increase the focus on early detection and management of NCDs, while still maintaining current efforts to manage infectious diseases.

## Supporting information

S1 Dataset The data set for the study is available as supplement. (XLSB)
